# Engineering Cofactor Preference of Ketone Reducing Biocatalysts: A Mutagenesis Study on a γ-Diketone Reductase from the Yeast *Saccharomyces cerevisiae* Serving as an Example

**DOI:** 10.3390/ijms11041735

**Published:** 2010-04-14

**Authors:** Michael Katzberg, Nàdia Skorupa-Parachin, Marie-Françoise Gorwa-Grauslund, Martin Bertau

**Affiliations:** 1 Institute of Technical Chemistry and Biotechnology, Freiberg University of Mining and Technology, Leipziger Straße 29; 09596 Freiberg, Germany; 2 Department of Applied Microbiology, Lund University, Getingevägen 60, 22241 Lund, Sweden

**Keywords:** biocatalysis, 2,5-hexanedione, *S. cerevisiae*, site-directed-mutagenesis, Gre2p, cofactor preference

## Abstract

The synthesis of pharmaceuticals and catalysts more and more relies on enantiopure chiral building blocks. These can be produced in an environmentally benign and efficient way *via* bioreduction of prochiral ketones catalyzed by dehydrogenases. A productive source of these biocatalysts is the yeast *Saccharomyces cerevisiae*, whose genome also encodes a reductase catalyzing the sequential reduction of the γ-diketone 2,5-hexanedione furnishing the diol (2*S*,5*S*)-hexanediol and the γ-hydroxyketone (5*S*)-hydroxy-2-hexanone in high enantio- as well as diastereoselectivity (ee and de >99.5%). This enzyme prefers NADPH as the hydrogen donating cofactor. As NADH is more stable and cheaper than NADPH it would be more effective if NADH could be used in cell-free bioreduction systems. To achieve this, the cofactor binding site of the dehydrogenase was altered by site-directed mutagenesis. The results show that the rational approach based on a homology model of the enzyme allowed us to generate a mutant enzyme having a relaxed cofactor preference and thus is able to use both NADPH and NADH. Results obtained from other mutants are discussed and point towards the limits of rationally designed mutants.

## 1. Introduction

Enantiopure chiral alcohols and especially those with more than one functional group or stereocenter are of increasing importance as building blocks for the synthesis of chiral pharmaceuticals, flavors and agrochemicals, as well as homogeneous catalysts [1–3]. A representative example among the multitude of chiral alcohols is the diol (2*S*,5*S*)-hexanediol ((2*S*,5*S*)-**1**). It is a key building block in the synthesis of chiral ligands used in phospholane based, homogeneous transition metal catalysts like (*R*,*R*)-Me-DuPhos-Rh which have proven useful in a number of asymmetric hydrogenation reactions [3–5]. Furthermore the chiral diol (2*S*,5*S*)-**1** is also of use in the synthesis of a variety of chiral pharmaceutical intermediates, agrochemicals and chiral auxiliaries [3,6–9].

In order to satisfy the demand for this chiral building block an efficient synthetic route yielding enantiopure (2*S*,5*S*)-**1** is needed. Although numerous synthetic strategies have been developed in the past including asymmetric hydrogenation with Ru-BINAP [10], multistep syntheses [4,11,12] and enzymatic resolution of racemates [13,14], the most efficient strategy is direct reduction of prochiral 2,5-hexanedione (**3**) mediated by dehydrogenases and microorganisms harboring these enzymes, respectively. Biocatalytic reduction of ketones to obtain chiral alcohols is not only efficient in terms of atom economy [15] but also highly selective and environmentally friendly, as it operates under mild reaction conditions [16,17].

**Scheme 1.**
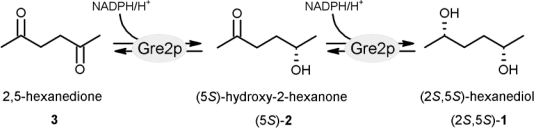
Reduction of 2,5-hexanedione (**3**) is stereospecifically catalyzed by Gre2p, yielding the chiral building blocks (5*S*)-hydroxy-2-hexanone ((5*S*)-**2**) and (2*S*,5*S*)-hexanediol ((2*S*,5*S*)-**1**) in a consecutive reaction.

The first biocatalyst reported of being capable of stereoselectively reducing 2,5-hexanedione was the baker’s yeast *S. cerevisiae* [18]. Although two other biocatalysts, catalyzing the same reaction with comparable stereospecificity, namely the dehydrogenases ADH-T from *Thermoanaerobacter* sp. [19] and ADH-A from *Rhodococcus ruber* [20], have been reported since then, the dehydrogenase responsible for 2,5-hexanedione reduction in *S. cerevisiae* was identified only recently as the gene product of GRE2 [21]. The latter enzyme - designated Gre2p - is especially interesting when it comes to the production of the monohydroxyketone (5*S*)-hydroxy-2-hexanone ((5*S*)-**2**) which is, just like (2*S*,5*S*)-**1,** a valuable chiral building block [22]. However (5*S*)-**2** is far more difficult to obtain than (2*S*,5*S*)-**1**, thus it is of significance that it arises as the intermediate in the consecutive (*S*)-selective reduction of **3** (Scheme 1) [23]. This intermediate transiently accumulates to up to 90% the amount of starting material when Gre2p is used as a catalyst, whereas usage of ADH-T and ADH-A, respectively yields lower amounts [23,24]. Hence the use of Gre2p is advantageous for the efficient production of enantiopure (5*S*)-**2**.

But the use of Gre2p as a biocatalyst is not restricted to reduction of **3**. The enzyme rather accepts a broad range of substrates including aliphatic and aromatic ketones, chloroketones, diketones as well as β-ketoesters which are reduced with high stereoselectivity [21,25–32]. The ability of Gre2p to accept several substrates is also reflected in studies dealing with its physiological role in *S. cerevisiae*, in which the enzyme is discussed to play a role in the catabolism and metabolism of quite different molecules such as ergosterol [33], isovaleraldehyde [34] and methylglyoxal [35].

It is this broad substrate range which makes Gre2p a highly valuable enzyme in biocatalysis. However if Gre2p is to be used for bioreductions *in vitro* additional care has to be taken for cofactor regeneration. Thereby it has to be noted that Gre2p as well as ADH-T and ADH-A prefer NADPH as a cofactor, which is compared to its alternative -NADH- less stable [36] and three times more expensive (according to the 2009 Sigma-Aldrich Catalogue). Hence in terms of biocatalysis a Gre2p variant preferring NADH would be more desirable.

In order to switch cofactor preference of an enzyme, mutagenesis is the method of choice. Especially site-directed mutagenesis has proven useful to alter the cofactor preference of a given dehydrogenase [37–41], in which context it has been reported that in some cases changing even one sole amino acid is sufficient to invert cofactor preference [41–44]. The latter was also achieved for enzymes which are grouped together with Gre2p into the family of short-chain dehydrogenases/reductase (SDR) [38,45–48]. Thus it can reasonably be assumed that an NADH-preferring variant of Gre2p should also be generable by just changing one amino acid. This is in accordance with the literature in which it is reported that within the SDR-family cofactor preference is thought to be determined by one amino acid [49]. Thus we were encouraged to design and generate a tailored Gre2p mutant, which preferably uses NADH as the cofactor of choice through exchanging just one amino acid by rationally guided site-directed mutagenesis. In the case of success this rational approach based on fundamental knowledge of enzyme architecture in SDRs would be much more efficient than generating tailored enzymes through “directed evolution”, which relies on labor intensive screening of a huge number of mutants [50]. However it has to be stated that in most cases knowledge on enzyme-activity-relationships is still too limited to rationally design a mutant having the expected properties which in turn renders “directed evolution” a technique yielding the expected results often faster than rational approaches. Nevertheless it should be the aim to gain so much understanding of enzyme architecture that rational creation of an effective and selective enzyme becomes possible. The results of this work contribute to the knowledge on enzyme-activity-relationships in SDRs and thus may take us a bit further to the ultimate goal of rationally designed enzymes.

## 2. Results and Discussion

In order to design a tailored enzyme insights into its structure are a prerequisite. Since a structural model based on X-ray crystallography does not yet exist for Gre2p, a structural model (homology model) based on a homologous dehydrogenase with known crystal structure needed to be constructed.

### 2.1. Homology Modeling of Gre2p

**Figure 1.**
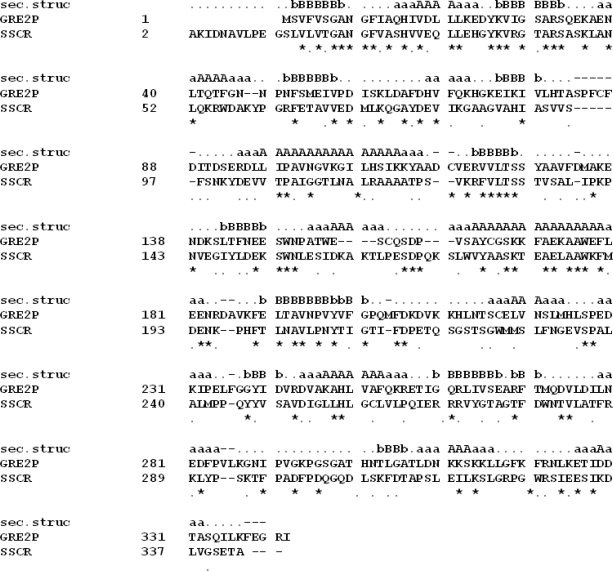
Amino acid sequence alignment of the *S. cerevisiae* dehydrogenase Gre2p (GRE2P) with *Sporobolomyces salmonicolor* carbonyl reductase (SSCR) upon which the Gre2p homology model was built. The secondary structure is also given (sec.struc), in which B/b indicates β-sheets and A/a α-helices. The last line indicates whether an amino acid is conserved between SSCR and Gre2p by using standard symbols.

Within the protein data bank (PDB; www.rcsb.org) the carbonyl reductase I from *Sporobolomyces salmonicolor* (SSCR; PDB-Code: 1Y1P) shares the highest sequence identity (29%) and similarity (18%) with Gre2p and thus serves as the models template (E-value = 3e^−22^; 96% of Gre2p aligned). On the basis of a global sequence alignment between Gre2p and SSCR (Figure 1) it is now possible to assign the secondary and tertiary structure taken from the template SSCR to the primary structure of Gre2p, thus constructing the desired homology model.

According to the overall sequence identity between model and template the quality and reliability of the model is in the so called “twilight zone”, in which false-positive results increase if a secondary structure is deduced from a sequence alignment [51,52].

Although the model may not reliably reflect the whole structure of Gre2p in detail, it will reliably reflect sub-parts of the enzyme, sharing a higher pair-wise sequence identity and similarity, with SSCR than the whole enzyme does. The latter is especially true for the N-terminal part of Gre2p which shares 44% identity and 18% similarity with SSCR on the sequence level.

**Figure 2.**
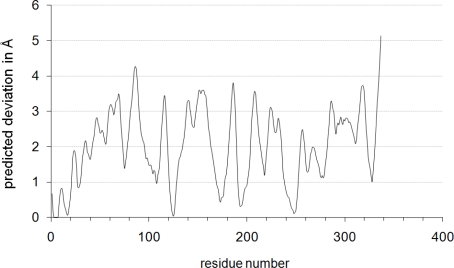
Predicted deviation of every amino acid residue in the Gre2p homology model as assessed by the QMEAN scoring function. QMEAN returns the estimated error of the space coordinates per amino acid residue in Ångström. More reliable regions are characterized by a residue error below 1 Å, whereas potentially unreliable regions have an error of >3.5 Å. It is evident from the figure that the N-terminal region (residues 0–50) of the modeled protein structure can be assessed to be quite reliable since the mean predicted deviation in this region is 1.3 Å.

Besides local sequence alignments, the local quality of a homology model can also be described through scoring functions such as ProQres [53] or QMEAN [54] (Figure 2). Both algorithms indicate that the homology model of Gre2p reliably describes its N-terminus. This is of outstanding importance since the N-terminal part of Gre2p, SSCR and other short-chain-dehydrogenases, is responsible for binding and recognizing the nicotinamide cofactor *via* the so called Rossmann-fold [55–57], which is exactly the target region to be altered by mutagenesis.

### 2.2. Structural Basis of Cofactor Specificity in Gre2p

**Figure 3.**
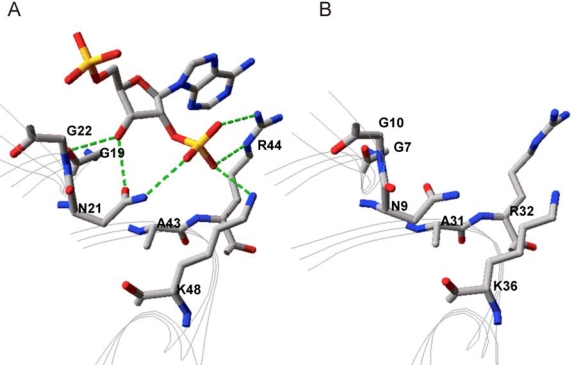
(**A**) Binding of the 2′-phosphate group attached to NADPH’s adenyl ribose moiety in SSCR (data from crystal structure PDB-Code: 1Y1P) [42]. (**B**) This binding motif is conserved in Gre2p (data from homology model).

By placing NADP^+^ of 1Y1P into the theoretical structure of Gre2p *in silico* a first insight into its binding could be obtained. Since it is our aim to change the cofactor preference of the enzyme it is especially important how the dehydrogenase is able to discriminate between NADH and NADPH. As can be seen from Figure 3 Gre2p and SSCR both share a common principle for discriminating NADPH from NADH and preferring the former. Just like it was proposed for the binding of NADPH by SSCR [58] Gre2p most probably binds the NADPH 2′-phosphate through electrostatic interactions with an arginine and lysine residue (Arg32, Lys36 in Gre2p; Arg44 and Lys48 in SSCR). As such, formation of salt bridges between the negatively charged phosphate group and the positively charged amino acid side chains stabilizes binding of NADPH, whereas NADH, due to the lack of a negatively charged phosphate group at the 2′-position of adenyl ribose, is not able to undergo such a strong interaction and hence is not able to bind as tightly as NADPH.

Apart from Arg32 and Lys36 there is an additional amino acid, Asn9, which is in close proximity to the adenyl ribose 2′-phosphate group and thus may stabilize its binding through hydrogen bonds with the 3′-hydroxyl-group and 2′-oxygen atom of the adenyl ribose moiety (Figure 3).

**Figure 4.**
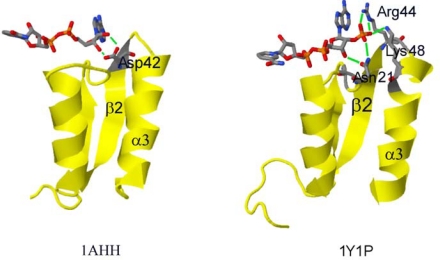
Structural basis of cofactor discrimination in dehydrogenases having a Rossmann-fold. Depicted are sections of the NADH preferring 7α-hydroxysteroid dehydrogenase from *E. coli* (left, PDB-Code 1AHH) and the carbonyl reductase from *Sporobolomyces salmonicolor* (right; PDB-Code 1Y1P). Possible hydrogen bonds are indicated by green lines.

The described phosphate group binding pattern is consistent with the hypothesis of NADPH binding in the family of short-chain-dehydrogenases/reductases (SDR) [49,56,57] to which SSCR and Gre2p belong [46,58]. According to this theory, SDRs in general bind cofactors *via* a Rossmann-fold [55,59] in which an amino acid having a basic side chain (in most cases Arg, some cases Lys; Arg32 in Gre2p) located in the loop after the second β-sheet (β_2_α_3_-turn, counted from N-terminus of protein) confers preference for NADPH. Alternatively this basic amino acid can also be located in the Rossmann-fold specific, so called “glycine rich motif”, in which in most of the cases an amino acid having a hydrophilic side chain such as Asn, Gln, Ser, Thr (Asn9 in Gre2p) is found [49,57] (Figure 4). With the latter considerations in mind it should be possible to rationally design a Gre2p mutant with altered cofactor specificity on the basis of the developed Gre2p homology model.

### 2.3. Design and Experimental Evaluation of Mutants

As it is reported that cofactor discrimination in SDRs largely depends on whether a basic amino acid (Arg, Lys) or an acidic amino acid (Glu, Asp) is found in the β_2_α_3_ loop in the cofactor binding Rossmann-fold [49] (Figure 4), it should be possible to interconvert the cofactor preference of a given SDR just by changing this one amino acid. In the case of Gre2p this would mean exchanging Arg32 with Glu and Asp, respectively. The negatively charged side chain of Glu32 and Asp32, respectively would then lead to the electrostatic repulsion of the negatively charged adenine ribose 2′-phosphate group of NADPH thus constraining its binding. Furthermore the carboxy group of Asp or Glu could form two hydrogen bonds with both 2′ and 3′-hydroxy-groups of the adenine ribose of NADH, as such resembling the situation in NADH preferring dehydrogenases (1AHH in Figure 4).

**Figure 5.**
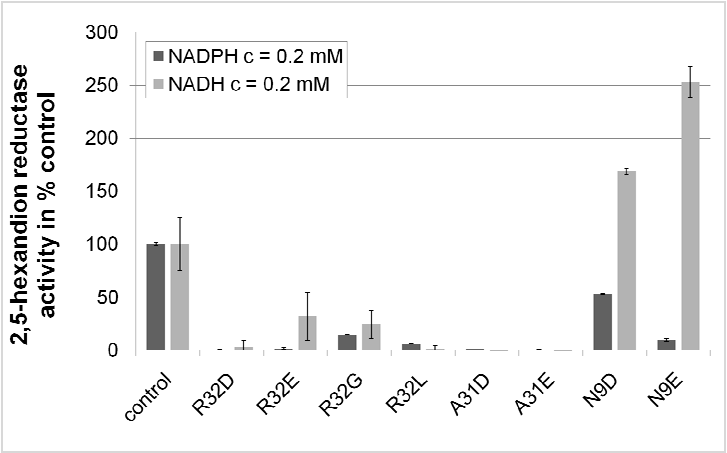
Impact of mutations in the N-terminal region of Gre2p on its 2,5-hexanedione reducing activity. Activities are normalized to the wild-type parent = 100% (control), in which the NADPH-dependent 2,5-hexanedione reductase activity [140 U/g (protein)] is almost 25 times higher than the NADH-dependent one [6 U/g(protein)]. This value is changed to 0.9 in the mutant N9E indicating that this mutant slightly prefers NADH over NADPH.

In practical Gre2p variants in which Arg32 was replaced by Glu or Asp indeed showed a markedly reduced activity with NADPH as a cofactor (Figure 5), which underlines the importance of Arg32 for binding of NADPH. However compared to the wild-type enzyme NADH-dependent activity was also reduced, rendering this approach ineffective for generating a Gre2p variant preferring NADH over NADPH.

The loss of activity in short-chain dehydrogenases upon replacement of an arginine analogous to Arg32 in Gre2p has also been reported for salutaridine dehydrogenase from *Papaver somniferum* [60] as well as for human 17-β-hydroxysteroid dehydrogenase, which suggests that there is a common reason for this effect. As has been proposed by McKeever *et al.* [61] arginine can play a dual role. Accordingly, Arg32 in Gre2p would not only be able to establish salt bridges or hydrogen bonds with the 2′-phosphate group of the cofactors adenine ribose moiety but also stabilizes binding of its adenyl moiety through π-π-stacking- and cation-π-interactions [62]. From this follows that the exchange of Arg32 against Glu or Asp will not only lead to electrostatic repulsion of NADPH but also to a weaker binding of adenine cofactors in general.

Besides that, the negative charge in the side chain of Glu32 and Asp32, respectively seems to further weaken binding of NAD(P)H cofactors, since substituting Arg32 against Gly and Leu restores some of the enzymes NADPH-dependent activity. This observation furthermore shows that π-stacking- and cation-π-interactions between Arg32 and the cofactors adenyl-moiety are not indispensable for cofactor binding in Gre2p, although the lack of these interactions remarkably decreases the enzymes activity.

In case of Gre2p Arg32Leu NADPH binding may be facilitated through hydrophobic interactions between Leu32 and the cofactor’s adenyl-moiety, whereas glycine at position 32 may increase flexibility of this part of the protein and thus could enable Gre2p to adopt a conformation which allows for more effectively binding the cofactor.

Nevertheless the conclusion has to be drawn that Arg32 is essential for cofactor binding in Gre2p and other SDRs. As such, substitution of Arg32 in Gre2p and homologous SDRs is not a promising approach for generating an NADH-preferring variant of any of these enzymes.

**Figure 6.**
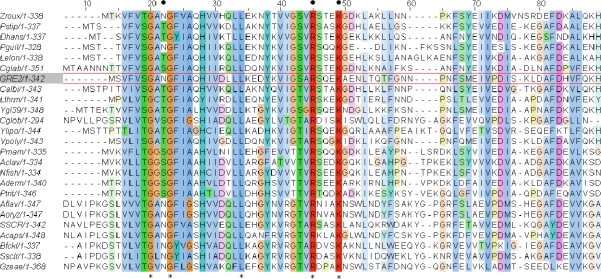
Multiple sequence alignment of proteins homologous to Gre2p from *S. cerevisiae* (highlighted in grey; red frame). Conserved amino acids are marked with an asterisk below the alignment, whereas residues thought to be involved in binding NADPH’s 2′-phosphate group are indicated by a dot above the alignment. For a full list explaining the abbreviations used see Table 1.

Furthermore, as Arg32 is conserved in this part of the protein among Gre2p homologs from other fungi (Figure 6), the observations made with Gre2p Arg32Gly,Leu,Asp,Glu underline that conserved amino acids are most often crucial for enzyme function and activity. Thus their sole substitution without changing other parts of an enzyme is likely to result in a mutant enzyme having inferior activity, when compared to its wild-type parent. Consequently further mutations only concerned non-conserved amino acids. Thus mutation of Lys36 in Gre2p, which is well conserved (Figure 6) and assumed to be involved in binding the 2′-phosphate group of NADPH (Figure 3), was not considered for mutation.

But in order to decrease the affinity of the enzyme for NADPH a negative charge in this phosphate binding region of Gre2p is a prerequisite. Thus the amino acid upstream of Arg32 (Ala31) was chosen as the next target.

**Figure 7.**
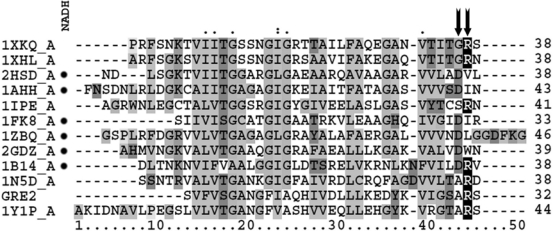
Sequence alignment of NADPH and NADH preferring short-chain dehydrogenases / reductases with known crystal structure labeled with its corresponding PDB Code. The N-terminal amino-acid-sequence is shown, which is responsible for cofactor recognition and binding. NADH preferring SDRs are assigned a dot after their respective PDB code (for a full table linking the PDB codes with information on the respective enzyme, see Table 2). Arrows are indicating those amino acids in the β_2_α_3_-turn which according to [49] determine the cofactor preferred by the enzyme.

Substituting Ala31 is expected to be successful as this yields an enzyme whose sequence resembles that of the *Drosophila* alcohol dehydrogenase, an enzyme which is reported to prefer NADH (1B14_A in Figure 7). Introducing an amino acid with an acidic side chain downstream of R32 (S33) is not reasonable, since the resulting enzyme would resemble the structure of the NADPH dependent porcine carbonyl reductase (1N5D_A in Figure 7) and thus is not likely to exhibit a changed cofactor preference.

Although exchanging amino acids equal to Ala31 in Gre2p successfully changed cofactor preference from NADPH to NADH in other SDRs like mouse lung carbonyl reductase [63] and human estrogenic 17-β-hydroxysteroid dehydrogenase [38], no change in cofactor preference could be observed in Gre2p when Ala31 was replaced by Asp and Glu, respectively. Instead both enzyme variants showed a significant loss of activity regardless of the cofactor used what clearly disqualifies substitution of Ala31 as a strategy for altering cofactor preference in Gre2p. Unfortunately this suggests that mutagenesis strategies are not transferable unrestrictedly from one enzyme to another even if they are homologous and grouped in one family.

**Figure 8.**
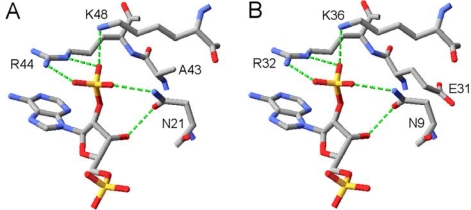
Possible orientation of Glu31 in the homology model of Gre2p Ala31Glu (**B**) compared to the situation in the crystal structure of SSCR (**A**). In both dehydrogenases the side chain of the amino acid residue upstream of arginine (Ala43 in SSCR; Glu31 in Gre2p Ala31Glu) points away from the cofactors ribosyl 2′-phosphate moiety and is thus not likely to interact with it.

One reason for the observed behavior may be the bonding angels (Φ/Ψ) in Ala31’s peptide bond. Assumed that both bonding angels Φ/Ψ in the homology model of Gre2p match the ones in the template SSCR (PDB: 1Y1P) and remain unchanged after substitution of Ala31 with Glu or Asp, the side chain of the amino acid at position 31 would not point towards the 2′-phosphate group of the cofactors adenine ribose moiety but in the opposite direction (Figure 8).

Thus the side chains of neither Glu31 nor Asp31 would be able to adopt a conformation allowing interaction of the side chains carboxy group with the 2′- and 3′-hydroxygroup of the cofactor’s adenyl ribose moiety. Instead the side chains would affect other parts of the protein yielding an almost inactive protein. Regardless of the underlying reasons, whose final clarification would require the crystal structure of Gre2p being solved (which is beyond the scope of this contribution) it has to be noted that substitution of Ala31 in Gre2p is unsuitable for generating an NADH dependent variant of the enzyme.

Far better results were obtained by exchanging Asn9 by Asp and Glu, respectively. As is depicted in Figure 5 substitution of Asn9 with Asp brought about a decrease in the NADPH dependent activity while at the same time NADH-dependent 2,5-hexanedione reductase activity increased. This difference becomes even more pronounced if the side chain of the amino acid at position 9 is prolonged by one carbon atom, *i.e.*, substituting Asp9 with Glu.

**Figure 9.**
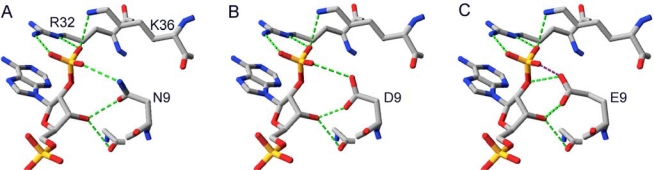
Proposed binding of NADPH in Gre2p wild-type (**A**) and its mutant variants Asn9Asp (**B**) and Asn9Glu (**C**). In Gre2p Asn9Glu NADH dependent 2,5-hexanedione reductases activity is increased, since Glu9 is able to form hydrogen bonds (green dotted lines) with both of the adenyl ribose hydroxyl groups and constrains binding of NADPH through electrostatic interaction as well as steric hindrance (possible collision with the phosphate moiety is indicated with a purple dotted line).

As Asn9 is already able to interact with the 3′-hydroxy and 2′-phosphate group of the cofactors adenine ribose moiety in the wild-type enzyme, it is reasonable that changing the properties of this amino acids side chain from polar but uncharged (Asn) to charged (Asp, Glu) brings about the electrostatic repulsion needed to decrease binding of NADPH and permits formation of hydrogen bonds between the 2′- and 3′-ribose hydroxyl groups and the side chain carboxyl group (Figure 9).

Compared to Gre2p Asn9Asp formation of hydrogen bonds and repulsion of NADPH’s additional phosphate group seems to become more pronounced in Gre2p Asn9Glu (Figure 5). This appears reasonable since the side chain of Glu is one carbon atom longer than the one of Asp and thus brings the carboxy group of Glu9 in closer proximity to the adenylribosyl 2′- and 3′-hydroxygroups, which allows for the establishment of strong hydrogen bonds between Glu9 and the cofactor. Moreover the side chain of Glu9 would even hamper binding of NADPH sterically (Figure 9).

The change of cofactor preference in Gre2p Asn9Glu is further corroborated by kinetic studies of this mutant enzyme. As is shown in Table 3 the mutants apparent Michaelis-constant for NADH is decreased by a factor of 1.5 compared to the wild-type parent. Similarly the apparent maximal velocity of the 2,5-hexanedione reduction using NADH increased almost 2-fold upon exchange of Asn9 with Glu. Thus cofactor preference of Gre2p was changed from strongly preferring NADPH in the wild-type to quite promiscuous in the N9E mutant which has no pronounced preference NADH and NADPH, respectively (Table 3).

As the enzyme is almost as stable as its wild-type parent (the half-live in 0.1 M phosphate buffer pH 7.0 at 30 °C was determined to be 24 h for the wild-type and 21 h for the Asn9Glu mutant) it may already be used to reduce 2,5-hexandion *in vitro* using NADH furnishing (5*S*)-**2** and (2*S*,5*S*)-**1**. The stereoselectivity of Gre2p [21,23] is unlikely to be altered by the exchange of Asn9 since the substrate binding pocket is about 15 Å away from Asn9 and made of amino acids far more downstream.

**Figure 10.**
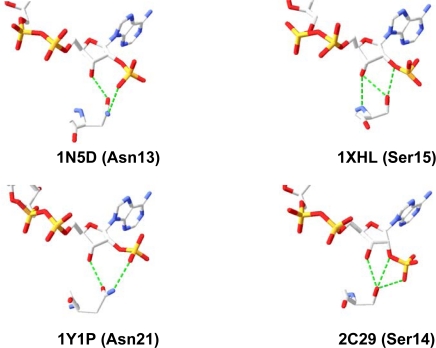
The amino acid residue corresponding to Asn9 in Gre2p is a promising target for alteration of cofactor preference in NADPH-dependent SDRs in general, since this amino acid is already forming hydrogen bonds with the 2′-phosphate and 3′-hydroxy group of the cofactor’s adenyl ribose moiety. Depicted is the orientation of this certain amino acid towards the 2′-phosphate and 3′-hydroxy group attached to the cofactor’s adenyl ribose moiety taken from four representative crystal structure of NADPH dependent SDRs. 1ND5–carbonyl reductase from *Sus scrofa* [64]; 1XHL–putative tropinone reductase II from *Caenorhabditis elegans* [65]; 1Y1P–carbonyl reductase from *Sporobolomyces salmonicolor* [58]; 2C29–dihyroflavonol reductase from *Vitis vinifera* [66].

Hence there is substantial evidence that exchanging Asn9, which is situated within the so called “glycine rich motif” of Gre2p, with Asp or Glu improves the enzyme’s ability to use NADH as a cofactor. Furthermore this observation appears likely to be not restricted to the single case of Gre2p. Since a hydrophilic amino acid residue (namely Asn, Thr, Ser *etc.*) corresponding to Asn9 in Gre2p, is found in many NADPH-dependent SDRs (see Figure 6 and [57]), it can be suggested that exchanging Asn9 with Glu and Asp, respectively, will - in most of the cases - result in an improved capability of the enzyme to use NADH as a cofactor. From crystal structures of NADPH-dependent SDRs is furthermore known that this certain amino acid often adopts a conformation which allows formation of hydrogen bonds between this residue and the cofactor’s 2′-and 3′-hydroxy groups attached to the adenyl ribose moiety (for a few examples see Figure 10).

Hence exchanging it with Glu and Asp, respectively will most likely lead to an enzyme in which binding of NADH is improved in the same way as is reported for the mutant Gre2p Asn9Glu (Figure 9).

Taken together this study allows concluding that cofactor preference in Gre2p is not only determined by the presence of an amino acid with basic or acidic side chain in the loop after the β_2_-strand. Apparently preference for NADPH is particularly conferred by the presence of a second amino acid with a basic side chain (Arg, Lys) found at least three amino acids downstream of the conserved Arg residue (e.g., in the SDR 2C29 Arg37 is separated from Lys44 by six amino acids).

Thus an inversion of cofactor preference in Gre2p can only be obtained if several amino acids are exchanged. For instance it would be of interest whether an effective enzyme preferring NADH can be obtained through exchanging the full loop after the sheet β2 with the one found in NADH dependent SDRs.

However a single substitution of one amino acid is sufficient to broaden cofactor acceptance and usage. Thus this study has shown that in SDRs amino acids within the conserved GxxG Motif are a valuable target for mutations if cofactor preference is to be altered.

Besides the success of having generated a Gre2p mutant with relaxed cofactor preference it must be noted that this mutant enzyme does not reach the ideal of being as catalytically efficient using NADH as it is the wild-type using NADPH. As such the mutant enzymes K_M_(NADH) is still more than 20 times higher than the K_M_(NADPH) of the wild-type Gre2p and the ratio v_max_(NADPH)_wt_/v_max_(NADH)_N9E_ is significantly greater than one, although the ultimate goal would be to keep this ratio at a value of one or below.

Similar observations were also made in previous rational mutagenesis studies aiming for alteration of cofactor preference in short-chain dehydrogenases [38,47,48]. For instance Huang *et al*., inverted the cofactor preference of human estrogenic 17-β-hydroxysteroid dehydrogenase by replacing Leu36 with Asp (Leu36 corresponds to Ala31 in Gre2p). But also in this case the mutant is not as catalytically efficient as the parent (30 times smaller catalytic efficiency), which is also reflected in the reported Michaelis-constants. As such the mutants (L36D) K_M_(NADH) is 17 times bigger than the K_M_(NADPH) of the corresponding wild-type enzyme [38].

Thus it appears that although a switch or at least a relaxation in cofactor preference of a short-chain dehydrogenase is achievable by merely mutating one single amino acid residue, the resulting enzyme will most probably not have the same catalytic efficiency as its wild-type parent. This result is rather disadvantageous if the enzyme of choice is to be used in a biotechnological process. Since high space time yields (∼100 g/L*d) and low biocatalyst loading are clear prerequisites for economically viable biotransformations [67], mutagenesis is expected to provide enzymes which exhibit the desired properties, with a catalytic efficiency being equal to or preferentially exceeding that of their wild-type counterparts. In order to achieve this by rational approaches, far too less is known about enzyme architecture and structure activity relationships in whole enzymes. Thus from a current point of view methods using random mutagenesis and high-throughput screening appear more promising than rational approaches in generating new powerful tailored biocatalysts. However with an increasing knowledge in enzyme architecture and structure activity relationship rational design of tailor made enzymes appears not only feasible but also much less time-consuming than multiple rounds of diversity generation and successive screening.

## 3. Experimental Section

### 3.1. Chemicals

Technical grade 2,5-hexanedione was obtained from Wacker Chemie AG (Munich, Germany) and purified to >99% by distillation. DNA-modifying enzymes were purchased from Fermentas (St. Leon-Rot, Germany).

#### 3.1.1. Strains and Growth Conditions

Within this study plasmid propagation was carried out with *Escherichia coli* TOP10 (Invitrogen™, Calrlsbad, CA, USA), whereas *E. coli* BL21 (DE3) pLysS (Invitrogen™) was used for production of recombinant proteins. All strains generated were stored at −80 °C as glycerol stocks (15% glycerol in LB-medium). Cultivation of *E. coli* strains was carried out at 37 °C in LB (lysogeny broth)-medium (1% bacto-tryptone; 0.5% yeast extract; 1% NaCl; pH 7.5) containing the appropriate antibiotics (*E. coli* TOP10 strains: 100 μg/mL ampicillin; E. coli BL21 strains: 100 μg/mL ampicillin + 35 μg/mL chloramphenicol). Medium was solidified with 1.8% agar agar when needed.

In order to produce plasmid encoded proteins in *E.coli* BL21(DE3) pLysS, fresh LB-medium was inoculated with pre-grown (LB-media; 37 °C, overnight) cells yielding an OD_620_ of 0.2. The growth of the culture at 30°C was monitored until the OD_620_ had reached 0.7. At this point IPTG was added up to a final concentration of 1 mM. The cultivation was continued for another 3 h before the cells were harvested by centrifugation (3,220 × g; 10 min), washed (with water and with 0.1 M potassium phosphate buffer) and subjected to disintegration.

#### 3.1.2. Molecular Biology Techniques/Construction of Plasmids

Cloning and modification of DNA was carried out using standard techniques as described by [68]. Plasmids were isolated using the GeneJet MiniPrep Kit from Fermentas. In order to construct an *E. coli* strain capable of Gre2p expression, the GRE2 gene was subcloned from p423-GPD-YOL151w (HIS3) [69] into pRSET B (Invitrogen) after introducing the restriction sites PstI and HindIII. The resulting plasmid was named pRSET B GRE2.Site-directed mutations were introduced into the gene of interest by using an adapted Quikchange™ protocol. Therefore a 50 μL PCR-reaction contained 0.5 μg of template (pRSET B GRE2), 200 μM dNTP’s, 0.18 μM of each primer (Table 4) and 2.5 U Pwo-Polymerase (Roche). After a 5 min initiation phase at 94 °C to denature template DNA, amplification of the desired plasmid was achieved with 18 cycles of the following temperature regime: 94 °C (0.5 min); 55 °C (0.5 min); 68 °C (6 min) followed by a final 10 min elongation phase at 68 °C. The success of the PCR could be verified by agarose-gel electrophoresis of 15 μL of the product mixture. In order to destroy the template 20 μL of PCR product were treated with 1 μL of DpnI FastDigest™ (Fermentas) enzyme for 1 h at 37 °C. Finally 1 μL of the DpnI treated and untreated PCR-reaction-product, respectively was transformed into electrocompetent *E.coli* TOP10 cells by electroporation. After cultivation on LB-agar overnight 2 colonies were picked for propagation, isolation and sequencing of the plasmid if the number of colonies arising form cells transformed with untreated PCR-product exceeded the number of colonies arising from cells transformed with DpnI treated PCR-product, by a factor of ∼10.

### 3.2. Homology Modeling

The three-dimensional structural model of Gre2p was constructed through homology modeling using the crystal structure of carbonyl reductase from *Sporobolomyces salmonicolor* (PDB: 1Y1P) as a template. In brief a sequence alignment between GRE2 and 1Y1P was generated under due consideration of the templates secondary structure with Clustal X2 (profile alignment mode, standard parameters) [70]. The alignment was optimized manually by using the software swiss pdb-viewer (SPDV) [71] Version 4.0.1, with the “mean force potential” [72] of each amino acids serving as the optimization criteria. After removal of clashes in the protein structure by SPDV, the final homology model was automatically built by the Swiss Model Server [73]. The latter also allows for assessing the quality of the obtained model through different scoring functions such as QMEAN [54] and ProQres [53]. All figures showing enzyme structures were generated with SPDV Version 4.0.1 [71].

#### 3.2.1. Enzyme Assays

The activity of Gre2p towards the diketone 2,5-hexanedione was determined by measuring the decrease in absorbance of NAD(P)H at 340 nm with a spectrophotometer. If not stated otherwise, the standard assay in 0.1 sodium phosphate buffer pH 7.0 contained, the enzyme, 10 mM 2,5-hexanedione and 0.2 mM of the reduced cofactor. The reaction was started by addition of 2,5-hexanedione and monitored for 90 s. In terms of this assay 1 U corresponds to the formation and consumption, respectively of 1 μmol of cofactor per min at pH 7.0 and 30 °C.

#### 3.2.2. Disintegration of *E. coli* Cells

*E. coli* cells were disintegrated by adding the non-ionic detergent B-PER (Pierce) in twice the amount of the cells wet weight. After addition of DNAse (Promega; 1 U/μL; v_DNAse_ = 1/60 of the volume of B-PER) the mixture was shaken for 20 min. The cell crude extract was obtained after removing cell debris by centrifugation (10 min; 16,100 × g).

#### 3.2.3. Kinetic Characterization

Determination of an enzymes apparent Michaelis-constant K_M_ and the apparent maximal velocity v_max_ for the reduction of 2,5-hexanedione was carried out by using 2,5-hexanedione in saturation (10 mM) and varying the concentration of the cofactor within the range of 0.1–4 mmol. The constants were obtained through fitting the data to the Michaelis-Menten-equation (non-linear regression). Before use the recombinant enzyme was enriched from crude extracts of *E. coli* by treating the crude extract with 20% (NH_4_)_2_SO_4_ and removing the precipitate.

## 4. Conclusions

It is widely recognized that biocatalysis is an invaluable tool to produce chiral building blocks and other high value chemicals in an economically attractive and environmentally benign way. However in order to be applied in an industrially relevant process on a larger scale, biocatalysts often need to be tailored to the process requirements. One example is switching the cofactor preference of NADPH dependent dehydrogenases to NADH, the latter being more cost-effective and more stable than its phosphorylated counterpart. The method of choice to achieve this goal is mutagenesis, as it allows for tailoring the enzyme’s properties through either random or rational modification of its primary structure.

In this study we investigated the impact of rationally introduced mutations upon cofactor preference of the NADPH-preferring dehydrogenase Gre2p originating from *S. cerevisiae*, which proved to be a valuable biocatalyst such as the reduction of the diketone 2,5-hexanedione. As the products of this reaction [(5*S*)-hydroxy-2-hexanone and (2*S*,5*S*)-hexanediol] are important chiral building-blocks, Gre2p is of major economic relevance for industry. However in order to apply this process on an industrial scale, an NADH preferring variant of Gre2p would be favorable.

In theory substitution of one amino acid should suffice to invert cofactor preference. Although this has been reported to be true for a series of short-chain-dehydrogenases – an enzyme superfamily Gre2p belongs to – the situation appears more complex with this enzyme. However there were two promising enzyme variants among the set of mutants generated and tested for activity which lead to a relaxation of cofactor preference yielding a mutant which accepts NADPH as well as NADH for the reduction of 2,5-hexanedione. The obtained results suggest that the GxxG motif which is often found at the N-termini of short-chain dehydrogenases/reductases (SDR), is a promising target in general, if the cofactor preference of a SDR is to be altered. As the impact of introduced mutations on enzyme activity and selectivity is still not predictable reliably, more structure activity studies on enzymes are needed in order to achieve the ultimate goal of *de novo* design of tailored biocatalysts.

## Figures and Tables

**Table 1.** Abbreviations used in Figure 6.

**Abbreviation**	**Name/putative function of the protein**	**Source organism**	**NCBI GenBank Accession number**
Zroux	AF178079_1 dehydrogenase	*Zygosaccharomyces rouxii*	AAF22287.1
Pstip	GRE2 methylglyoxal reductase	*Pichia stipitis CBS 6054*	XP_001384081
Dhans	DEHA2A06314p hypothetical protein	*Debaryomyces hansenii*	CAG84554.2
Pguil	PGUG_03246 hypothetical protein	*Pichia guilliermondii ATCC 6260*	XP_001485517.1
Lelon	hypothetical protein	*Lodderomyces elongisporus NRRL YB-4239*	XP_001527072.1
Cglab	hypothetical protein	*Candida glabrata*	XP_445918.1
GRE2	Yol151wp / 3-methyl butanal reductase	*Saccharomyces cerevisiae*	NP_014490
Calbi	oxidoreductase	*Candida albicans SC5314*	XP_719286
Lthrm	KLTH0F04026p	*Lachancea thermotolerans CBS6340*	XP_002554383
Ygl39	dehydrogenase YGL039w	*Saccharomyces cerevisiae*	NP_011476.1
Cglob	CHGG_00287 hypothetical protein	*Chaetomium globosum CBS 148.51*	XP_001219508.1
Ylipo	YALI0D07062p	Yarrowia lipolytica	XP_502514
Vpoly	hypothetical protein	*Vanderwaltozyma polyspora DSM 70294*	XP_001642950.1
Pmarn	ketoreductase	*Penicillium marneffei ATCC 18224*	XP_002151938
Aclav	ketoreductase	*Aspergillus clavatus NRRL 1*	XP_001275327
Nfish	ketoreductase	*Neosartorya fischeri NRRL 181*	XP_001260510
Aderm	ketoreductase	*Ajellomyces dermatitidis SLH14081*	XP_002625096
Ptrit	dihydroflavonol-4-reductase	*Pyrenophora tritici-repentis Pt-1C-BFP*	XP_001938846
Aflav	aldehyd reductase II	*Aspergillus flavus NRRL3357*	EED56897.1
Aoryz	RIB40 hypothetical protein	*Aspergillus oryzae*	XP_001817435.1
SSCR	carbonyl reductase	*Sporobolomyces salmonicolor*	AAF15999
Acaps	aldehyd reductase	*Ajellomyces capsulatus G186AR*	EEH09765.1
Bfckl	BC1G_06734 hypothetical protein	*Botryotinia fuckeliana B05.10*	XP_001554946.1
Ssclr	SS1G_13307 hypothetical protein	*Sclerotinia sclerotiorum 1980*	XP_001585790.1
Gzeae	FG11217.1 hypothetical protein	*Gibberella zeae PH-1*	XP_391393.1

**Table 2.** Abbreviations used in Figure 7.

**PDB-Code**	**Name of the protein**	**Source organism**	**Reference**
1XKQ	short-chain dehydrogenase / reductase with unknown function	*Caenorhabditis elegans*	[74]
1XHL	tropinone reductase II	*Caenorhabditis elegans*	[65]
2HSD	3α,20β-hydroxysteroid dehydrogenase	*Streptomyces exfoliates*	[75]
1AHH	7 α-hydroxysteroid dehydrogenase	*Escherichia coli*	[76]
1IPE	tropinone reductase II	*Datura stramonium*	[77]
1FK8	3α-ydroxysteroid dehydrogenase	*Comamonas testosteroni*	[78]
1ZBQ	17-β-hydroxysteroid dehydrogenase 4	*Homo sapiens*	[79]
2GDZ	15-hydroxyprostaglandin dehydrogenase type1	*Homo sapiens*	[80]
1B14	alcohol dehydrogenase	*Drosophila lebanonensis*	[81]
1N5D	carbonyl reductase	*Sus scrofa*	[64]

**Table 3.** Apparent kinetic constants determined for Gre2p wild-type (Gre2p wt) and its mutant Gre2p Asn9Glu (Gre2p N9E).

	**Gre2p wt**		**Gre2p N9E**	
	NADPH	NADH	NADPH	NADH
	
K_M_^c^in mM	0.038	1.45	1.52	0.96
v_max_^a^,^c^in U/g (protein)	288	81	272	147
Cofactor preference b	136	0,007	1.2	0.9

a v_max_ is given instead of k_cat_ since the protein was not purified to homogeneity;

b cofactor preference can be assessed by dividing the enzymes’ catalytic efficiencies (k_cat_/K_M_) obtained with NADPH as a cofactor by the one obtained with NADH. As the amount of enzyme can be crossed out in the latter formula k_cat_ can be substituted by v_max_; values above zero indicate preference for this cofactor;

c all kinetic constants given are apparent constants since only the amount of the cofactor was varied, whereas the concentration of 2,5-hexanedione was kept constant at 10 mM.

**Table 4.** Primers used in this study.

**Name^a^**	**Sequence^b^**
GRE2N_F (PstI)^c^	5′–AACTGCAGAACAGATAGCAGTATCACACGCCCGTAAAT–3′
GRE2N_R (HindIII)^c^	5′–AAAAGCTTGAAGAGAAAAATGCGCAGAGATGTACTAGATGAT–3′
R32E_F	5′–GGTCATCGGTTCTGCC**GAA**AGTCAAGAAAAGGCCGAGAATTTAACGG–3′
R32D_F	5′–GGTCATCGGTTCTGCC**GAC**AGTCAAGAAAAGGCCGAGAATTTAACGG–3′
R32G_F (BamHI)	5′–GGTCATCGGTTCTGCC**GGA**TCCCAAGAAAAGGCCGAGAATTTAACGG–3′
R32L_F	5′–GGTCATCGGTTCTGCT**TTA**AGTCAAGAAAAGGCCGAGAATTTAACGC–3′
A31E_F	5′–GAAGACTATAAGGTCATCGGTTCT**GAG**AGAAGTCAAGAAAAGGCC–3′
A31D_F (BstYI)	5′–GAAGACTATAAGGTCATCGGATCT**GAT**AGAAGTCAAGAAAAGGCC–3′
N9D_F (BsgI)	5′–CAGTTTTTGTTTCAGGTGCA**GAC**GGGTTCATTGCCCAAC–3′
N9E_F	5′–CAGTTTTTGTTTCAGGTGCT**GAG**GGGTTCATTGCCCAAC–3′

a name of the primer is deduced from the amino acids changed in Gre2p; only forward primer given; reverse primer corresponds to the reverse complement of the forward primer;

b nucleotides in the primer being responsible for amino acid exchange in Gre2p are printed in bold face; restriction sites are underlined;

c – Primer for introduction of restriction sites (given in parenthesis; underlined in sequence) flanking the cloned GRE2 gene.
